# Functionality of the GAL4/UAS system in *Tribolium *requires the use of endogenous core promoters

**DOI:** 10.1186/1471-213X-10-53

**Published:** 2010-05-19

**Authors:** Johannes B Schinko, Markus Weber, Ivana Viktorinova, Alexandros Kiupakis, Michalis Averof, Martin Klingler, Ernst A Wimmer, Gregor Bucher

**Affiliations:** 1 Ernst Caspari Haus, Georg-August-University Göttingen, Justus-von-Liebig-Weg11, 37077 Göttingen, Germany; 2 Department of Biology, Friedrich-Alexander-University Erlangen, Staudtstr. 5, 91058 Erlangen, Germany; 3 Institute of Molecular Biology and Biotechnology (IMBB), Foundation for Research and Technology Hellas (FoRTH), GR-70013 Iraklio Crete, Greece; 4 Max Planck Institute of Molecular Cell Biology and Genetics, Pfotenhauerstr. 108, 01307 Dresden, Germany; 5 Centre for Organismal Systems Biology, Faculty of Life Sciences, University of Vienna, Althanstrasse 14, 1090 Wien, Austria

## Abstract

**Background:**

The red flour beetle *Tribolium castaneum *has developed into an insect model system second only to *Drosophila*. Moreover, as a coleopteran it represents the most species-rich metazoan taxon which also includes many pest species. The genetic toolbox for *Tribolium *research has expanded in the past years but spatio-temporally controlled misexpression of genes has not been possible so far.

**Results:**

Here we report the establishment of the GAL4/UAS binary expression system in *Tribolium castaneum*. Both GAL4Δ and GAL4VP16 driven by the endogenous heat shock inducible promoter of the *Tribolium hsp68 *gene are efficient in activating reporter gene expression under the control of the Upstream Activating Sequence (UAS). UAS driven ubiquitous tGFP fluorescence was observed in embryos within four hours after activation while *in-situ *hybridization against tGFP revealed expression already after two hours. The response is quick in relation to the duration of embryonic development in *Tribolium *- 72 hours with segmentation being completed after 24 hours - which makes the study of early embryonic processes possible using this system. By comparing the efficiency of constructs based on *Tribolium, Drosophila*, and artificial core promoters, respectively, we find that the use of endogenous core promoters is essential for high-level expression of transgenic constructs.

**Conclusions:**

With the established GAL4/UAS binary expression system, ectopic misexpression approaches are now feasible in *Tribolium*. Our results support the contention that high-level transgene expression usually requires endogenous regulatory sequences, including endogenous core promoters in *Tribolium *and probably also other model systems.

## Background

The red flour beetle *Tribolium castaneum *has become established as an important model system, from a group of insects - the Coleoptera - that comprises one fourth of all described animal species [1] including numerous pests (boll weevil, corn rootworm, Colorado potato beetle and Asian longhorn beetle). While the technical amenability of the fruit fly *Drosophila melanogaster *remains unmatched, there are topics which cannot be readily addressed in the fly. On one hand, evolutionary questions require comparative functional data from several species. Moreover, many processes are derived in *Drosophila *and data from more insect typical taxa is needed. For instance, segments are specified all at one time in *Drosophila *(long germ mode) instead of sequential formation that is characteristic of most insects (short germ mode); embryonic legs do not develop in *Drosophila *while insect larvae usually do have functional walking appendages; extraembryonic membranes are highly reduced and the head is involuted during embryogenesis in *Drosophila*, resulting in seemingly headless lavae [2,3]. On the other hand, certain issues of insect biology cannot be studied in *Drosophila *because it lacks the respective character. One example are the odoriferous defensive glands that play a crucial role in insect communication and defense but are not found in *Drosophila*. Hence, there is a need for complementary insect model systems for comparative functional work and for studying processes that are difficult or impossible to study in *Drosophila*.

Recent development of genetic techniques has rendered *Tribolium *the second best insect model system. Its genome is sequenced [4], germ line transformation in *Tribolium *is as efficient as in *Drosophila*, and several marker and transposon systems for gene transfer are available [5-8]. Based on these systems, an insertional mutagenesis system has been established [9] which has been used to generate a collection of enhancer trap and homozygous lethal lines [10]. Most notably, robust RNAi techniques have been established. RNAi can be applied by embryo injection but the systemic uptake of dsRNA also allows injection of female pupae or adults and analysis of the embryonic phenotype in the offspring. Injection of larvae allows to uncover phenotypes during metamorphosis without interfering with earlier (embryonic) gene functions [11-18]. Apparently, all tissues can be targeted by RNAi [14] and the null mutant phenotype can be phenocopied by RNAi in many cases [19].

While knock-down of gene function via RNAi is extremely efficient in *Tribolium*, spatio-temporally controlled misexpression of genes has not been possible so far. Binary expression systems have the advantage that any gene can be expressed in tissue-specific patterns and at certain developmental stages - depending on the availability of driver lines [20-26]. This allows the study of dominant lethal or sterility inducing genetic constructs because the transgene is only activated when the driver and responder activities are combined. One widely used binary expression system is the GAL4/UAS system, which consists of a driver construct, where expression of the heterologous transactivator GAL4 is driven by an inducible or tissue specific enhancer. In the responder construct, the gene of interest is under the control of the heterologous GAL4-controlled Upstream Activating Sequence (UAS) [20,27-29]. For driver and responder, separate transgenic lines are generated and upon crossing these strains, the gene of interest is expressed in the progeny in the pattern defined by the driver.

GAL4 was identified in the yeast *Saccharomyces cerevisiae *as a regulator of *GAL1*, *GAL10 *and other genes induced by galactose [30,31]. GAL4 regulates transcription by binding to a 17 bp site in the UAS [32]. The GAL4 transactivator consists of two functional domains. The DNA binding domain maps to the first 74 amino acids whereas the activation domain maps to two regions, amino acids 148-196 and 768-881. In the transactivator version GAL4Δ, the activation domain is directly fused to its DNA binding domain[33,34]. This results in a smaller protein which has been shown to activate reporter gene expression about twice as effectively as the original GAL4 in *Drosophila *[23].

In the GAL4-VP16 version, the activation domain of GAL4 has been replaced by the highly acidic portion of the herpes simplex virus protein VP16 that activates transcription of immediate early viral genes [35-40]. It was shown that GAL4-VP16 can efficiently activate transcription in mammalian cells [41]. Also in *Drosophila *GAL4-VP16 is more active than GAL4, but it has been shown to be less useful compared to GAL4Δ given that many insertions of GAL4-VP16 constructs appear to be non-functional [23].

After its inception in the mouse [29], the Gal4/UAS system has been established in *Drosophila *where it has become a standard technique adapted to diverse uses [42]. One of the numerous extensions of this system is its combination with GAL80, a protein that binds to the carboxy-terminal amino acids of GAL4 and inhibits activation of transcription [23,27-29,42-45]. The GAL4/UAS binary system has been adapted to silkworm *Bombyx mori *[46] zebrafish [47], *Xenopus *[48] and *Arabidopsis *[49]. With this work we establish the GAL4/UAS system in *Tribolium*. We show that both GAL4Δ and GAL4-VP16 transactivate well, with GAL4Δ being slightly more efficient. Importantly, we find that the use of *Tribolium *endogenous core promoters is essential for efficient expression of transgenes.

## Methods

### Constructs

**Figure 1:**
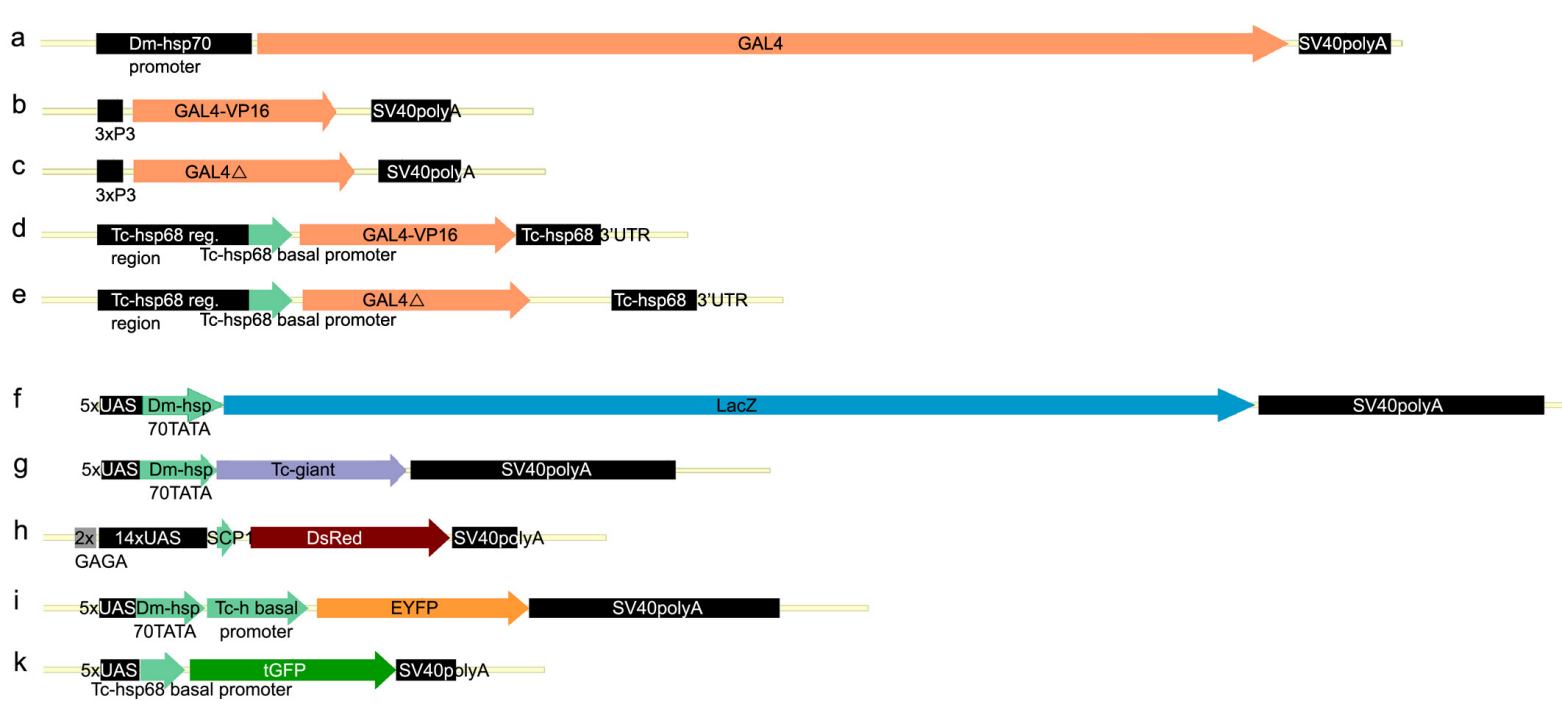
**Schematic representation of driver and responder constructs**. Driver constructs: (**a**) Dm-hs-GAL4; (**b**) 3 × P3-GAL4-VP16; (**c**) 3 × P3-GAL4Δ; (**d**) Tc-hsp-GAL4-VP16; (**e**) Tc-hsp-GAL4Δ. Responder constructs: (**f**) UAS-Dm-hsp-LacZ; (**g**) UAS-Dm-hsp-Tc-giant; (**h**) UAS-SCP1-DsRed; this construct contains GAGA sites to prevent position effects. (**i**) UAS-Dm-hsp-Tc-bh-EYFP; (**k**) UAS-Tc-bhsp-tGFP.

All transactivator and responder constructs were stably integrated into the genome by transposition using the *piggy*Bac vectors pBac[3xP3-EGFPafm], pBac[3xP3-ECFPafm] [50], pBac[3 × P3DsRedaf] [6] or pXL-BacII[3 × P3-EYFPaf], which was generated in a series of minimal *piggyBac *constructs along with pXL-BacII[3 × P3-EGFPaf], pXL-BacII[3 × P3-ECFPaf], and pXL-BacII[3 × P3-DsRedaf] by exchanging the *Eco*RI-*Eco*RV fragment of pXL-BacII-ECFP [51] with an EcoRI-NruI fragment carrying the fluorescent marker and the restriction sites *Asc*I and *Fse*I [6,50]. However, the integration efficiency of pXL--BacII derivatives appears to be lower than with the other version containing more piggyBac sequence (not shown).Transactivator plasmids: pBac[3 × P3-EGFP;Dm-hs-GAL4]; pBac[3 × P3-EYFP;3xP3-GAL4-VP16] [23]; pBac[3xP3-EYFP;3xP3-GAL4Δ] [23]; pBac[3xP3-EGFP;Tc-hsp5'-GAL4-VP16] [GenBank acc no GU452684]; pBac[3 × P3-EGFP;Tc-hsp5'-GAL4] [GenBank acc no GU452683]. Responder plasmids: p[51] Bac[3 × P3-Dsred;UAS-Dm-hsp70TATA-LacZ] [23]: pBac[3 × P3-DsRed;UAS-Dm-hsp70TATA-Tc'giant]; pBac[3 × P3-EGFP;gUAS-SCP1-DsRed]; pXL-BacII[3 × P3-DsRed;UAS-Dm-hsp70TATA-Tc'h-EYFP]; pBac[3 × P3-DsRed;UAS-Tc-bhsp68-tGFP] [GenBank acc no GU452685]; Heat shock plasmid: pBac[3XP3-DsRed;Dm-hsp70-EGFP] [52]. The sequences of those constructs that were functional are available at genbank. Sketches of the constructs are depicted in fig. 1, detailed maps are available from the authors. Dm: *Drosophila melanogaster*, Tc: *Tribolium castaneum*; hsp: heat shock promoter; bhsp: basal heat shock promoter/core promoter.

### Tribolium stocks and germline transformation

*Tribolium *germline transformation was performed according to standard procedure by injecting *piggy*Bac constructs (500 ng/μl in injection buffer, i.e. 5 mM KCl, 0.1 mM KH_2_PO_4_, 0.1 mM Na_2_HPO_4 _pH 6.8) into embryos of the *vermillion*^*white *^(*v*^*w*^) strain [5,7,53], together with 300 ng/μl helper plasmid phspBac [54]. A Femto Jet (Eppendorf, Hamburg, Germany) device with pulled and cut borosilicate glass capillaries was used for injections. Injected embryos were kept under humid conditions for two days at 32°C/90°F, afterwards transferred to lower humidity and kept at 32°C/90°F until they hatched. Larvae were collected and transferred to full wheat flour. Adult G0 beetles were crossed to *v*^*w *^wild type animals, and transgenic beetles were outcrossed again with *v*^*w*^.

### Transformation markers and epifluorescence microscopy

As transformation markers, EGFP [55,56] (Clontech Laboratories Inc., Palo Alto, CA), EYFP [57], ECFP [58] as well as the humanized variant DsRed1 [6] were used. The 3 × P3-driven expression pattern of the fluorescent markers was detected in the eyes of *T. castaneum *using a Leica MZ 16FA fluorescence stereomicroscope with a planachromatic 0.8 × objective (Leica, Wetzlar, Germany). Filter sets used were specifically designed EGFP-LP (Ext. 470/40; Emm. 500lp), ECFP-LP (Ext. 425/50; Emm. 460lp), and DsRedwide (Ext. 546/12; Emm. 605/75).

### *Tribolium *crosses

To activate the binary expression system, adult beetles selected for the dominant markers of the driver and responder lines, respectively, were crossed together and kept for 10 days at 28°C/81°F. Within this period of time, sperm from previous matings will be largely replaced by that of the newly added males. Subsequently, the crosses were transferred to fresh flour for egg collections at 32°C/90°F. Transheterozygous embryos, larvae, pupae or adults were heat shocked and analyzed for tGFP fluorescence.

### Heat shock conditions

Embryonic heat shocks were performed in 1.5 ml Eppendorf tubes in a water bath for 10 minutes at 46°C/115°F. Larval, pupal and adult heat shocks were performed in 2 ml Eppendorf tubes for 20 minutes at 46°C/115°F. For activation of the *Drosophila *constructs in *Tribolium *the animals were incubated 1h at 48°C in an incubator.

### Detection of tGFP fluorescence in embryos

After the heat shock, eggs were allowed to recover at 32°C (see results section for respective recovery times and then dechorionated under mild conditions in Natriumhypochlorite (1% DanKlorix), and subsequently aligned on a microscope slide and analyzed for tGFP (Evrogen, Moscow, Russia) fluorescence every hour (Leica MZ 16FA fluorescence stereomicroscope with a planachromatic 0.8 × objective; EGFP-LP filter set, 30 × magnification, 10 sec. exposure time). These steps were performed at room temperature.

### Detection of reporter gene expression by in situ hybridization

For comparison of reporter gene expression, eggs from 0-72 hour collections at 25°C/77°F were heat shocked and fixed 11 hours later. Whole-mount *in-situ *hybridizations were performed as described before [59] with probes of similar size and concentration (tGFP: 770 bp; 220 ng/μl; DsRed: 740bp, 230 ng/μl; eyfp: 790 bp, 220 ng/μl; lacZ: 750 bp, 230 ng/μl). Staining time was the same for all *in-situ *hybridizations.

## Results

### *Drosophila *constructs fail to work consistently in *Tribolium*

*Tribolium *beetles transgenic for fluorescent proteins under the control of the artificial 3xP3 enhancer-promoter element exhibit strong fluorescence in larval, pupal and adult eyes as well as parts of the nervous system [5]. Similar expression is found in a variety of other arthropods [5,50,60-66]. Moreover, several *Drosophila *constructs have been shown to work in other species [46,52]. Hence, our first approach to introduce the GAL4/UAS system in *Tribolium *was to directly transfer constructs tested in *Drosophila *[20,23] to *Tribolium*. We used both the transactivator versions GAL4Δ and GAL4-VP16 (Fig. 1b, c) driven by the 3xP3 enhancer-promoter [23]. Transgenic beetles for each of these constructs were crossed to beetles carrying a responder with *lacZ *under the control of UAST (Fig. 1f) or UASp (not shown) [20,28]. Due to an endogenous ß-galactosidase-like activity in the beetle eyes (not shown) we could not use an enzyme activity assay to detect the *lacZ*-reporter. By Western blot we did not detect ß-galactosidase in transheterozygotes while Alpha-tubulin was detected. The positive control from an extract of *D. melanogaster *heads expressing *lacZ *via a functional LexAGAD/*(LL)4 *system [23] was readily detected (Additional file 1A). We also tested a responder construct with *Tc-giant *[67] under the control of UAST (Fig. 1g) with a driver consisting of Gal4 under the control of the *Drosophila *heat-shock promoter (Fig. 1a). We expected phenotypes similar to those elicited by heat-shock induced *giant *misexpression in *Drosophila *namely the loss of at least four segments from first instar cuticles [68]. The analysis of heat shocked offspring for cuticle phenotypes did not reveal significant differences to controls that had not been subjected to heat shock (not shown). In conclusion, our attempts to directly transfer *Drosophila*-based constructs to *Tribolium *failed.

### GAL4Δ and GAL4-VP16 activate reporter gene expression via endogenous core promoters

Core promoters might influence the efficiency of transcription in a species specific way. In order to test if the failure in the above experiments was due to the use of *Drosophila *core promoters, we made a set of constructs using *Tribolium *specific core promoters in both driver and responder constructs. We tested two versions of transactivators, GAL4Δ and GAL4-VP16 [34,41], both driven by the endogenous heat shock inducible *Tc-hsp68 *promoter element (JBS and GB, unpublished) which contains HSF binding sites similar to those found in *Drosophila *heat inducible promoters (Fig. 1d, e). This promoter leads to strong ubiquitous transcription within 10 minutes after heat shock in embryos. However, earliest blastoderm stages are refractory to heat shock induced expression while shortly before the morphological differentiation of extraembryonic from embryonic tissue the response is strong (GB, unpublished). The responder, turboGFP (tGFP) was used as reporter, fused to the SV40 early mRNA polyadenylation signal, and driven by UAS sites placed upstream of a 150 bp fragment containing the basal (non-heat-shock-responsive) *Tc-hsp68 *promoter (Fig. 1k). This core promoter does not drive expression on its own but can do so when combined with enhancer elements (JBS and GB, unpublished). In order to exclude position effects, we analyzed two independent transgenic lines for each construct, i.e. four activator lines were each crossed to two responder lines. Self crossed UAS responders were included as negative controls. A 24 hour egglay was collected and heat shocked (see materials and methods), and then checked for fluorescence every hour. Fastest expression of tGFP in embryos could be observed using the UAS-Tc-bhsp-tGFP#2 line in combination with both GAL4Δ driver lines, while the GAL4-VP16 lines took about one hour longer before tGFP fluorescence could be detected (Table 1). The UAS-Tc-bhsp-tGFP#7 line tended to be activated later than the #2 line, indicating some position effect. But also with the #2 reporter line, GAL4Δ tended to perform better than GAL4-VP16. On average, when crossing the two different responder lines to the GAL4Δ lines, tGFP fluorescence was visible 3.5 hours after heat shock, whereas in crosses with the GAL4-VP16 lines 4.5 hours were necessary for first detection (Table 1).

**Table 1:** Comparison of GAL4Δ and GAL4-VP16

	UAS-Tc-bhsp-tGFP	
	#2	#7
Tc-hsp-GAL4Δ #1	3 h	4 h
Tc-hsp-GAL4Δ #2	3 h	4 h
Tc-hsp-GAL4-VP16 #2	4 h	4 h
Tc-hsp-GAL4-VP16 #3	4 h	6 h

**Response to GAL4Δ is slightly faster than to GAL4-VP16**. Shown is the time when first fluorescence of tGFP was visible after heat shock. The driver lines Tc-hsp-GAL4Δ#1 and #2 as well as Tc-hsp-GAL4-VP16#2 and #3 were crossed against the responder lines UAS-Tc-bhsp-tGFP#2 and #7. Combinations containing driver lines based on GAL4Δ result in earlier responder gene expression (3.5 hours on average) than combinations containing GAL4-VP16-based lines (4.5 hours on average).

### GAL4/UAS is widely applicable in different tissues and stages of *Tribolium*

**Figure 2:**
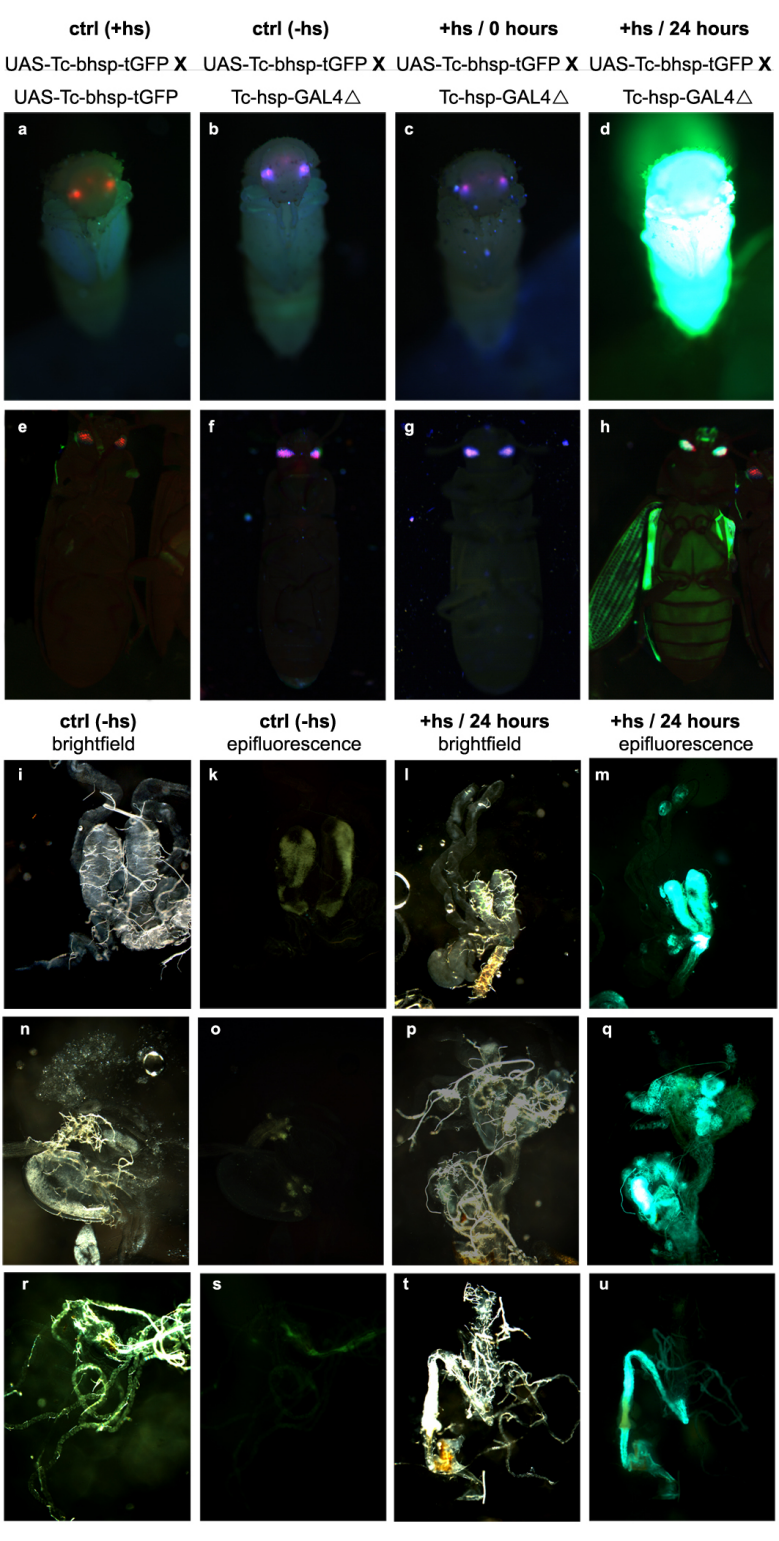
**The GAL4/UAS system is active in several *Tribolium *stages and tissues**. Animals positive for both, the GAL4Δ driver and UAS responder construct, show strong tGFP fluorescence after heat shock. Shown are pupae (a-d) and adults (e-h). (**a, e**) No fluorescence is visible in the negative control (heat-shocked animals that carry only the responder line UAS-Tc-bhsp-tGFP#7, i.e. eyes with red fluorescence only). (**b, f**) Without heat shock, animals carrying driver (Tc-hsp-GAL4Δ#1) and responder (UAS-Tc-bhsp-tGFP#7) - eyes marked red and blue - do not show body fluorescence. (**c, g**) Immediately after heat shock, animals carrying driver and responder construct exhibit no fluorescence of tGFP. (**d, h**) The same animals 24 h after heat shock exhibit strong tGFP fluorescence. Note expression in the adult wings (**h**). (**i-u**). Fluorescence is detected in some internal organs upon activation of the system (**m,q,u**) but not before heat shock (**k, o, s**; note low level of autofluorescence at different wave length). Tested were male reproductive organs (**i-m**), female reproductive organs (**n-q**) and gut (**r-u**). (i, l, n, p, r, t) are the respective bright field pictures.

In order to test the potential of the GAL4/UAS system for stage or tissue specific expression in *Tribolium*, we performed heat shocks in larvae, pupae and adult beetles. We observed tGFP fluorescence in animals carrying both the GAL4Δ and UAS constructs 24 hours after the heat shock treatment, at all stages tested (Fig. 2d, h), whereas immediately after the heat shock no fluorescence was detected (Fig. 2c, g). As negative controls served pupae and adults carrying both constructs which were not exposed to heat shock (Fig. 2b, f) and heat-shocked adults carrying either the transactivator (not shown) or the responder construct alone (Fig. 2a, e). These controls did not show increased fluorescence compared to wild type (Fig. 2). The same was true for larvae (data not shown). This experiment suggests that the GAL4Δ system is active in the beetle at all postembryonic stages. We further analyzed several tissues of adult animals for reporter activity 24 h after heat shock, and found the system to be active in all tissues examined including the wings (Fig. 2h), male and female reproductive organs (Fig. 2m and 2q, respectively), as well as the gut (Fig. 2u). Because a ubiquitous Gal4 driver is currently not available, we were not able to determine if all cells are responsive to the system. Animals of the same genotype that were not subjected to heat shock did not exhibit fluorescence in any of these tissues (Fig. 2k, o, s).

### Endogenous promoters are required for efficient function of transgenes in *Tribolium*

**Figure 3:**
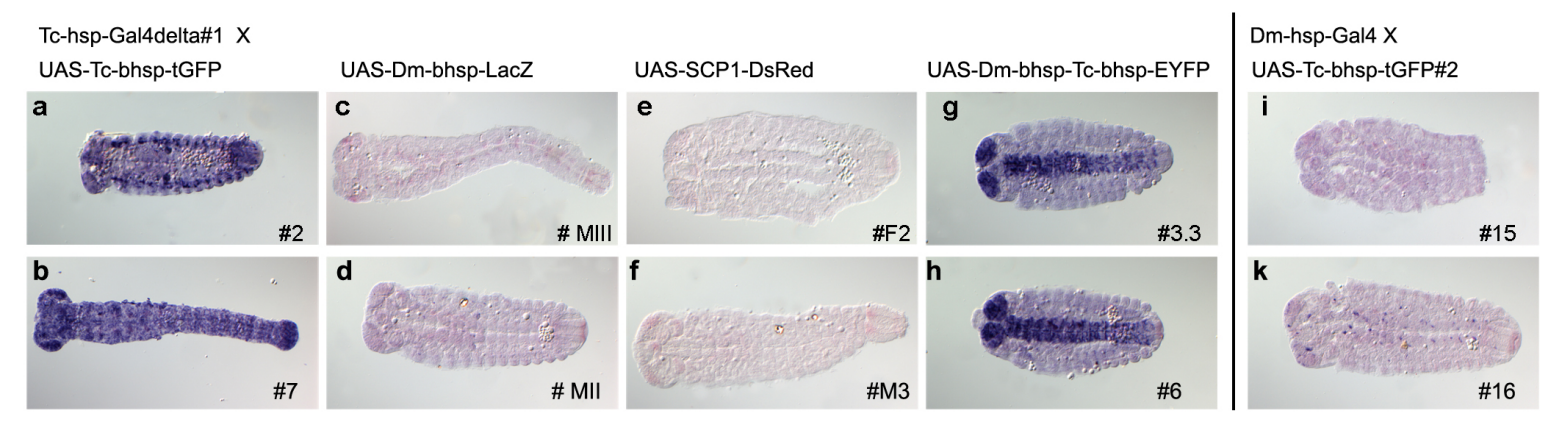
**Endogenous versus exogenous promoters in transactivator and responder constructs**. Only when *Tribolium *core promoters are used in the responder and transactivator constructs, activation of the reporter gene is observed via *in-situ *hybridization. Exogenous promoters are not capable of driving reporter gene expression. (**a-h**) Transactivator line Tc-hsp-GAL4Δ#1 was crossed to different responder lines and *in-situ *hybridization was performed with the respective antisense RNA probe. (**a, b**) In the positive controls (UAS-Tc-bhsp-tGFP) ubiquitous expression of tGFP is detected. In contrast, no reporter activity is detected in constructs utilizing a *Drosophila *core promoter (UAS-Dm-hsp-LacZ) (**c, d**) or an artificial "super core promoter" (UAS-SCP-DsRed) optimized for vertebrate cells (**e, f**). (**g, h**) A construct containing both the *Drosophila *basal heat shock promoter and the *Tribolium *hairy promoter (UAS-Dm-hsp-Tc-bh-EYFP) shows activation predominantly in the nervous system of advanced embryos. (**i-k) **The *Drosophila *heat shock promoter driving Gal4 shows no activity in one insertion line (**i**) while in the other some isolated cells show expression of the reporter (**k**).

These results suggest that the use of endogenous core promoters may be critical for the function of transgenes. To directly compare the relative efficiencies of different core promoters, we crossed the driver line Tc-hsp-GAL4Δ#1 to different responder lines which were based on non-*Tribolium *core promoters (Fig. 1f, g, h), induced GAL4Δ expression by heat shock and detected the transcript of the reporter gene by whole mount in situ hybridization. To achieve comparable staining levels, all probes were approximately the same size and concentration, and the stainings were developed for the same time. Two independent insertion sites for each responder construct were analyzed to control for integration site effects. As reference we used the above mentioned reporter lines UAS-Tc-bhsp-tGFP#2 and #7 were used (Fig. 3a, b).

First, we tested a responder construct containing the *Drosophila *basal *hsp70 *promoter (UAS-Dm-hsp-LacZ, Fig. 1f) which has been used successfully in *Drosophila *to drive *LacZ *[23]. We did not detect expression of *lacZ *mRNA in the offspring (Fig. 3c, d). Next, we tested the SCP1 core promoter which is an artificial core promoter that contains four core promoter motifs - a TATA box from the CytoMegaloVirus (CMV) gene IE1, a initiator (Inr) based on Adenovirus Major Late (AdML) genes and a *Drosophila *G retrotransposon, a motif ten element (MTE) from *Drosophila Tollo *and a downstream promoter element (DPE) from the *Drosophila *G core promoter - within a single promoter construct. It directs high levels of transcription by RNA polymerase II in nuclear extracts from *Drosophila *and HeLa cells, and is more efficient than the CMV or AdML core promoters [69]. This construct (Fig. 1h) contains additional GAGA elements to prevent position effects [70,71]. However, transgenic lines #F2 and #M3, carrying the gUAS-SCP1-DsRed construct (Fig. 1h), showed no expression of DsRed in embryos when crossed to our driver line and heat shocked (Fig. 3e, f). In order to test whether this failure is *Tribolium *specific, we generated two independent *Drosophila *lines using the same gUAS-SCP1-DsRed construct. These lines were crossed to the dpp-GAL4, ap-GAL4, da-GAL4 and ptc-GAL4 drivers and examined at larval stages. DsRed fluorescence was observed with the dpp-GAL4 driver, roughly consistent with the dpp pattern in discs, but not with the other drivers. Apparently, the vertebrate core promoter gUAS-SCP1 is not working well in insects. Together, these results indicate that neither a core promoter from *Drosophila *nor an artificial promoter optimized for vertebrate cells is efficient in driving expression in *Tribolium*.

Assuming that endogenous core promoters are required for efficient transcription, we tested another *Tribolium *core promoter. The *Tc-hairy *upstream region has been analyzed previously and from these data the putative *Tc-hairy *core promoter was deduced [72]. We added this putative basal *Tc-hairy *promoter (*Tc-bh*) just downstream of the Dm-hsp70TATA in the UAS-Dm-hsp-LacZ construct and exchanged the reporter *LacZ *with *egfp*. Hence, this construct (UAS-Dm-hsp-Tc-bh-EYFP, Fig. 1i) contains both *Drosophila *and *Tribolium *core promoters. When activated by GAL4Δ, strong expression of *eyfp *could be detected predominantly within the central nervous system in old embryos (Fig. 3g, h). At earlier stages expression was not efficient (not shown) and the negative control (UAS-Dm-hsp-Tc-bh-EYFP alone) did not exhibit detectable expression of *eyfp *in the absence of GAL4 transactivator activity (not shown). This surprising expression pattern was identical in two independently generated insertions of the same construct. We confirmed that *Tc-hairy *is not expressed in the central nervous system in embryos of this developmental stage (not shown). Hence, this unexpected restriction to the nervous system is probably not due to the integration site but either due to a hidden specific activity of the *Tc-hairy *core promoter, or due to interactions with cryptic binding sites elsewhere in the construct. Thus, although the Tc-hairy core promoter can confer strong expression in *Tribolium*, it may not be able to direct expression in all embryonic tissues.

In a similar way we also tested the efficiency of *Drosophila *promoters in the context of GAL4 drivers, in combination with the responder UAS-Tc-bhsp-tGFP #2. Dm-hs-GAL4 (Fig. 1a) utilizes the upstream region of the *Drosophila hsp70 *gene [73]. These heat shock constructs are frequently used in *Drosophila *(e.g. [54,74]). The transactivator line Dm-hs-GAL4 #16 induced weak expression of the reporter gene tGFP in single cells of advanced embryos (Fig. 3k) whereas with the transactivator line Dm-hs-GAL4 #15 no tGFP expression was detected (Fig. 3i). This indicates that the *Drosophila *heat shock promoter can elicit only weak Gal4 activity in a subset of *Tribolium *tissues which in addition may depend on the integration site.

To explore this further, we tested the ability of the *Drosophila hsp70 *promotor to directly drive EGFP in *Tribolium*. Studying four independent transgenic lines based on the construct pBac[3XP3-DsRed;Dm-hsp70-EGFP] that showed to be functional in the butterfly *Bicyclus anynana *[52] we found that the results were highly variable in *Tribolium*; two lines were capable of activating expression after prolonged heat shock in pupae (1hour at 48°C in an incubator; Additional file 1, Fig. S1 B, lines A and E), one was only mildly heat-inducible (Additional file 1, Fig. S1 B, line B) and one was active independent of heat-shock (Additional file 1, Fig. S1 B; line D). Some of these responses varied across developmental stages (see Additional file 1, Fig. S1 C). Taken together, we find that the *Drosphila *heat shock promotor does show some activity in *Tribolium *but its activity is weak and strongly influenced by position effects.

### Temperature dependence of the GAL4/UAS system

**Figure 4:**
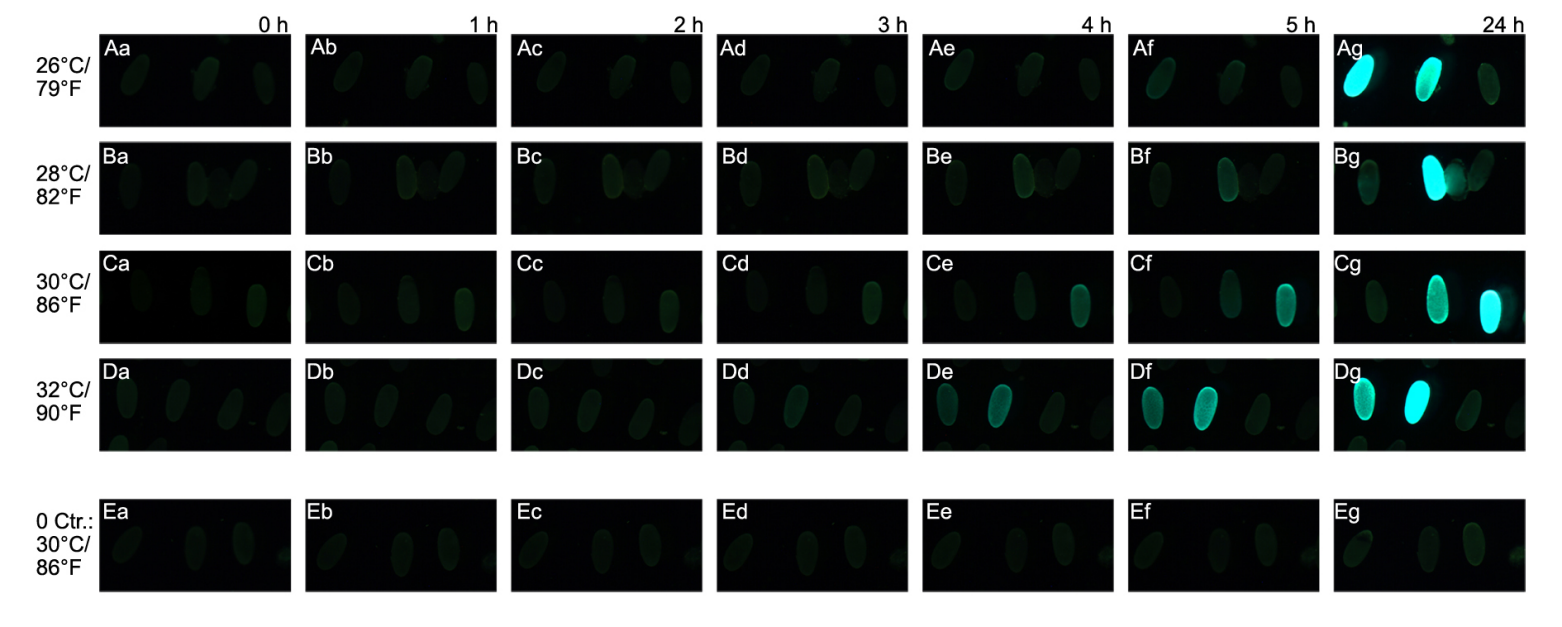
**Temperature dependence of the GAL4/UAS system in *Tribolium***. The transactivator line Tc-hsp-GAL4Δ#1 was crossed to the responder line UAS-Tc-bhsp-tGFP#7. Eggs from a 1h collection were heat shocked and checked for tGFP fluorescence directly after heat shock, at five one-hour intervals, and again after 24h. Each row shows the same embryos at a given time after heat shock kept at a certain temperature. As both lines are heterozygous, only about 25% of embryos were expected to exhibit tGFP fluorescence. In the bottom row, embryos of the heat shocked responder line at 30°C are shown as negative control. At 26°C, fluorescence is first seen 5 hours after heat shock (**Af**); at 28°C: 4-5 hours (**Be; Bf**); at 30°C: 3-4 hours (**Cd; Ce**); at32°C: 3 hours (**Dd**).

In *Drosophila*, it has been shown that GAL4 activity is enhanced in flies raised at 29°C/84°F compared to lower temperatures [42]. Therefore we wanted to test, whether this temperature dependence exists in *Tribolium *as well. 0 to 24 hours old *Tribolium *embryos derived from heterozygous parents carrying the transactivator Tc-hsp-GAL4Δ#1 and the responder UAS-Tc-bhsp-tGFP#7 were heat shocked, dechorionated, aligned on a microscope slide and kept at different temperatures. Pictures were taken directly after the heat shock and in one hour intervals to check for the onset of tGFP fluorescence. Indeed we found that higher temperatures lead to an earlier onset of fluorescence ranging from 5 hours (26°/79°F) to 3-4 hours (32°C/90°F) (Fig. 4). However, maximal tGFP activity (24h after heat shock) appears to be similar at both temperatures.

There is little difference in the kinetics of chromophore maturation at 28°C and 37°C for several GFP variants (this efficiency even decreases for some variants at higher temperatures [75]). Therefore, the earlier onset of tGFP fluorescence at higher temperatures could rather be due to higher GAL4Δ activity, due to faster kinetics of the gene regulatory machinery, or both. *Tribolium *development proceeds twice as fast at 32°C versus 26°C (total developmental time 6d vs. 3 d). In these experiments, maximal tGFP levels did not appear grossly different at these temperatures.

### The GAL4/UAS system is applicable in *Tribolium *embryogenesis

**Figure 5:**
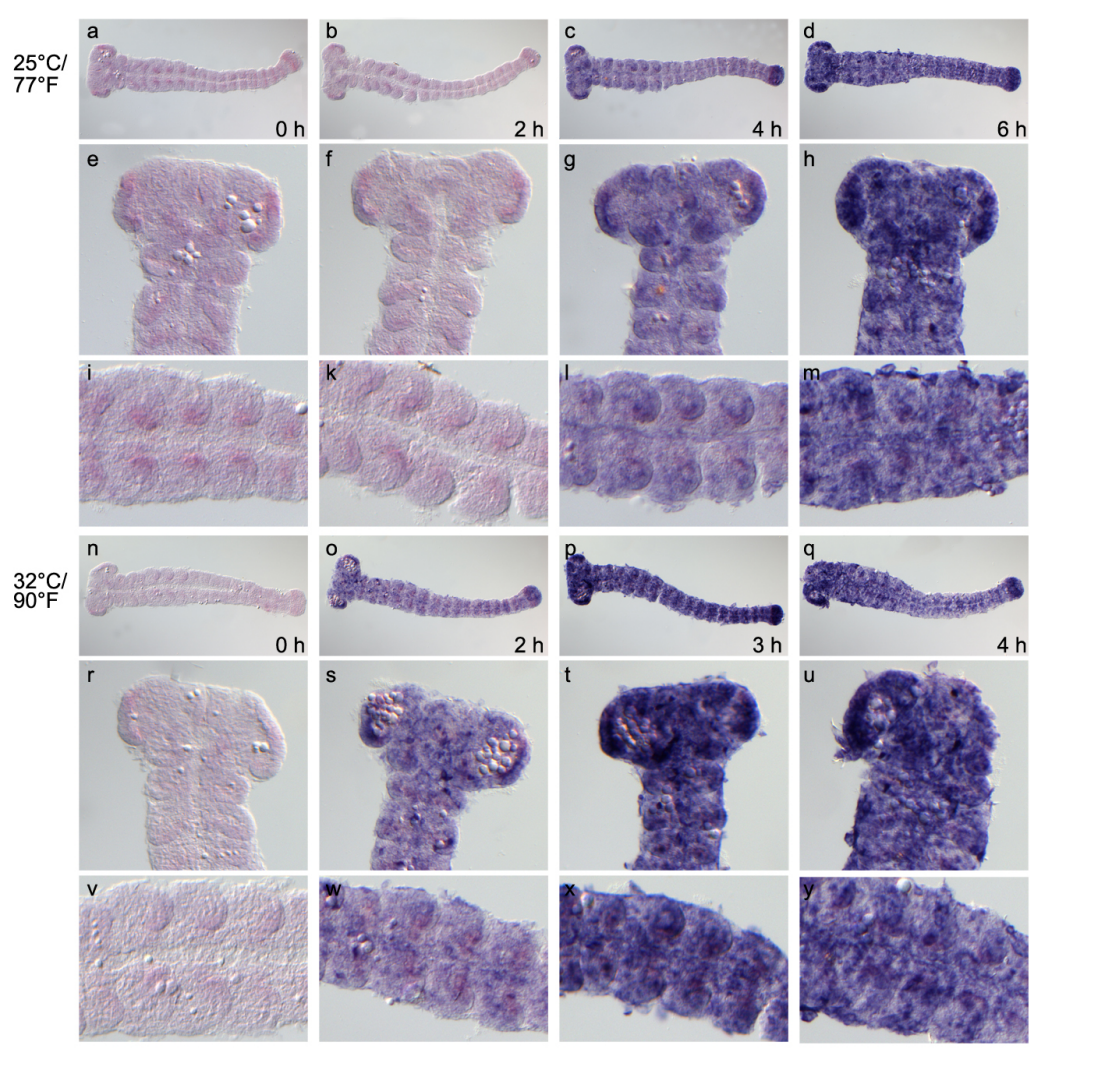
**Fast onset of GAL4 driven expression relative to embryonic developmental time**. Transheterozygous eggs were collected for one hour and aged for 18 hours before heat shock. They were then incubated at 25°C/77°F (**a-m**) or 32°C/90°F (**n-y**) respectively. At given time points, tGFP expression was detected by *in-situ *hybridization. (**a, b**) At 25°C, there is no expression of the reporter gene two hours after heat shock. (**c**) After 4 h weak expression is detected. (**d**) After 6 h strong expression of the reporter gene is seen. (**n**) At 32°C there is no expression directly after heat shock. The onset of expression is seen after 2 h (**o**) and remains high during the subsequent two hours (**p, q**). (**e-m**) and (**r-y**) show higher magnifications of the respective embryos above. Judged from head morphology and appendage outgrowth, embryonic development has progressed little between heat shock and first appearence of reporter gene expression (compare e, i to g, l and r, v to s, w).

Finally, we analyzed, whether the GAL4/UAS response is sufficiently rapid to analyze gene function during *Tribolium *embryogenesis. We tested how much time it takes from heat shock to expression of the reporter gene using the best combination of driver and responder lines (Tc-hsp-GAL4Δ#1 and UAS-Tc-bhsp-tGFP #7). A one hour egglay was collected and embryos were allowed to develop further until the end of germ band elongation. The embryos were then heat shocked and fixed for *in-situ *hybridization at different time points after the heat shock. It was unclear how temperature would affect the system - on the one hand embryonic development is about twice as fast at 32°C compared to 25°C, on the other hand the heat shock may slow down development and recovery might be aided by lower temperature. Hence, treated embryos were kept at 32°C or 25°C after heat shock. Embryos kept at 25°C were fixed immediately, or two, four and six hours after heat shock treatment (Fig. 5a-d). Embryos kept at 32°C were fixed directly after heat shock and after two, three and four hours, because faster onset was expected at higher temperatures (see above) (Fig. 5n-q). *tGFP *expression was detected via *in-situ *hybridization as an immediate readout of gene expression.

Earliest tGFP expression could be observed four hours after heat shock (10 min at 46°C) when incubated at 25°C, whereas embryos kept at 32°C after heat shock showed expression already after two hours (compare Fig. 5c to 5o). Strong expression was detectable already after three hours at 32°C while six hours were required for a similar expression level at 25°C (compare Fig. 5d to 5p). At the stages tested, the morphology of the head and the elongation of appendages provide a good measure for the age of the embryo. We compared overall morphology, head morphology and the length of the trunk appendages at the time of heat shock (Fig. 5e, i and 5r, v) and when the reporter was fully expressed (Fig. 5h, m and 5t, x, respectively) in at least 5 embryos per time point. We do not find a major difference indicating that the target gene response is fast relative to developmental time.

## Discussion

We have adopted the GAL4/UAS system to *Tribolium *and find that GAL4Δ [33,34] is slightly superior to GAL4-VP16 [41]. This came not unexpected as GAL4Δ is a much smaller protein that consists only of the DNA binding and activation domains of GAL4. As potential toxic effects of GAL4-VP16 have been observed for *Drosophila *[23,76] and in other organisms [41,77] we suggest the use of GAL4Δ in *Tribolium *in the future.

The GAL4Δ/UAS system is active at all stages of *Tribolium *development and activates reporter gene expression in a variety of different tissues indicating broad applicability. Interestingly, the activation of a UAS target is relatively fast compared to embryonic development which will allow the use of the system to investigate embryogenesis in addition to postembryonic stages. In fact, development does not seem to proceed much after heat shock (compare embryos d to a and q to n in Fig. 5). This is in line with findings in *Drosophila *where a 15' arrest of development has been observed upon heat shock [78]. Like in *Drosophila*, *Tribolium *embryos resume development after some time and do not show elevated lethality or cuticle phenotypes due to the heat shock conditions used (not shown). In contrast, the extremely fast mode of *Drosophila *early development has in many cases hampered the use of the Gal4/UAS system in the study of some processes. Furthermore, Gal4 is likely to be fully active at the temperatures commonly used to raise *Tribolium *(25-32°C) because they are similar to the temperature optimum for yeast (25-30°C) [79].

In contrast to endogenous core promoters, neither the *Drosophila *heat shock core promoter nor an artificial "super core promoter" (SCP1) consisting of optimized vertebrate core promoter motifs were effective in driving expression in *Tribolium*. Similar experience has recently been described for the tephritid fruit fly *Ceratitis capitata *[80,81]. On the other hand, *Drosophila *heat shock constructs have been shown to work in the silkworm *Bombyx mori *[46] and the butterfly *Bicyclus anynana *[52]. Moreover, the artificial 3xP3 enhancer is driving fluorescent reporters in the *Tribolium *eyes and brain using a *Drosophila *core promoter [5], and the same is true in a wide variety of other organisms [50,61-66]. In the light of our results it appears that the 3XP3 element is extremely efficient in driving expression and hence is able to override the poor function of the exogenous core promoter.

## Conclusions

The establishment of the Gal4/UAS system in *Tribolium *allows more profound functional gene analysis by directed expression in this species in the future. This will further promote *Tribolium *as a model organism where general biological questions can be studied. To take full benefit of the system, it is essential to generate a collection of driver lines that allow misexpression in different tissues. To this end it will be expedient to perform random insertion screens with GAL4Δ-containing mutator constructs following a procedure similar to the one recently used in the large scale GEKU insertional mutagenesis screen [10].

One important lesson for future transgenic tools in *Tribolium *and probably also other species is that the use of endogenous promoters is necessary for efficient expression. Even if constructs based on exogenous core promoters show some activity under certain circumstances or in some species, for full functionality, the use of species-specific promoters is essential.

## Authors' contributions

JBS made the constructs and the experiments using the endogenous *Tribolium *promoters and wrote the paper. GB designed this part of the study and wrote the paper. Constructs based on *Drosophila *core promoters and their tests in *Tribolium *were done by IV, MW, and GB under the guidance of EAW and MK. The gUAS-SCP1 construct and transgenic lines were contributed by AK and MA. All authors read and approved of the final version of the paper.

## Supplementary Material

Additional file 1 ***Drosophila *promoter does not work reliably in *Tribolium***. **A**) **Variants of Drosophila-based Gal4/UAS systems do not show any activity in *Tribolium castaneum***. UAS-dependent *LacZ *driven by a *Drosophila *core promoter (UAST) does not lead to detectable protein expression in adult heads (lanes 1 h/2 h, ten heads, respectively) or abdomen (1a/2a, one abdomen, respectively), when activated by 3xP3-driven GAL4Δ (lanes 1h/1a) or Gal4-VP16 (lanes 2 h/2a). Negative controls: UAST responder alone (lane 3) and *vermilion*^*white *^strain without transgenes (lane 4). Functionality of the anti-ß-galactosidase antibody was confirmed by an extract of *Drosophila *heads expressing *lacZ *by a functional LexAGAD/*(LL)4 *system ([23]; 3 heads used, lane 5). Additionally, reprobing of the blot with an anti-alpha-tubulin antibody was performed as a loading control. **B**) **The inducibility of the *Drosophila *heat shock promoter is low in *Tribolium *and its activity subject to position effect**. Four independent insertions of a construct with the *Drosophila heat shock 70 *promoter driving EGFP [52] were tested at the pupal stage (lines A, B, D and E). "+hs" indicates heat shocked animals. As controls, transheterozygotes without heatshock (-hs) and a heatshocked wt control (wt +hs) were included. Line A and E showed some activation at the pupal stage while little activation was observed in lines B and D. Moreover, line D showed some constitutive activity. **C**) **The inducibility of the *Drosophila *heat shock promoter is unreliable and partially stage-dependent in *Tribolium***. Heat shock activation of *Drosophila heat shock 70 *promoter driving EGFP [52] line A is strong only at the pupal stage. "+hs" indicates heat shocked animals. As controls, transheterozygotes without heatshock (-hs) and a heatshocked wt control (wt +hs) were included. Click here for file
