# Supplementary figures and images for: Functionality of the GAL4/UAS system in Tribolium requires the use of endogenous core promoters
Source: BMC Dev Biol. 2010 May 19;10:53. doi: 10.1186/1471-213X-10-53 (PMC2882914; doi:10.1186/1471-213X-10-53)

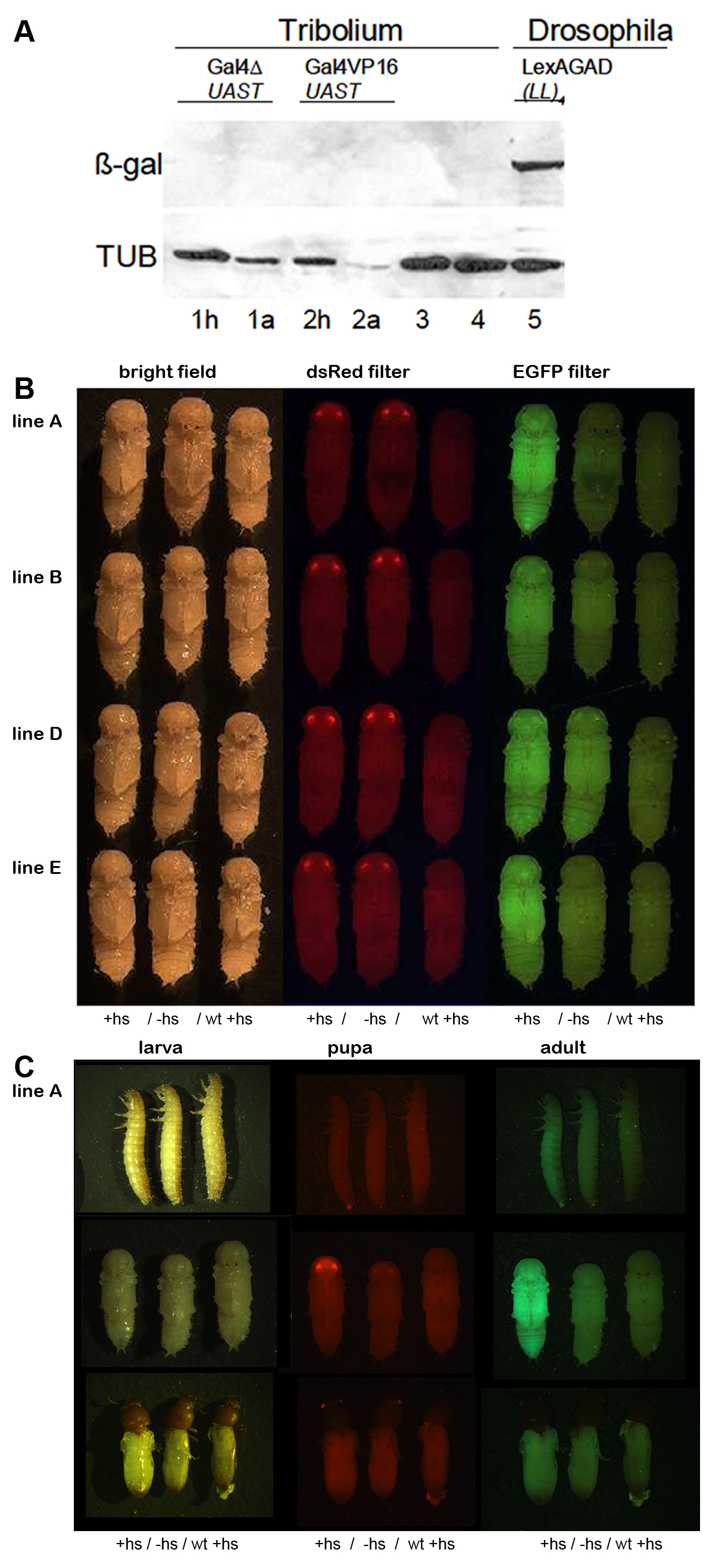

Supplement: Additional file 1 — Drosophila promoter does not work reliably in Tribolium. A) Variants of Drosophila-based Gal4/UAS systems do not show any activity in Tribolium castaneum. UAS-dependent LacZ driven by a Drosophila core promoter (UAST) does not lead to detectable protein expression in adult heads (lanes 1 h/2 h, ten heads, respectively) or abdomen (1a/2a, one abdomen, respectively), when activated by 3xP3-driven GAL4Δ (lanes 1h/1a) or Gal4-VP16 (lanes 2 h/2a). Negative controls: UAST responder alone (lane 3) and vermilionwhite strain without transgenes (lane 4). Functionality of the anti-ß-galactosidase antibody was confirmed by an extract of Drosophila heads expressing lacZ by a functional LexAGAD/(LL)4 system ([23]; 3 heads used, lane 5). Additionally, reprobing of the blot with an anti-alpha-tubulin antibody was performed as a loading control. B) The inducibility of the Drosophila heat shock promoter is low in Tribolium and its activity subject to position effect. Four independent insertions of a construct with the Drosophila heat shock 70 promoter driving EGFP [52] were tested at the pupal stage (lines A, B, D and E). "+hs" indicates heat shocked animals. As controls, transheterozygotes without heatshock (-hs) and a heatshocked wt control (wt +hs) were included. Line A and E showed some activation at the pupal stage while little activation was observed in lines B and D. Moreover, line D showed some constitutive activity. C) The inducibility of the Drosophila heat shock promoter is unreliable and partially stage-dependent in Tribolium. Heat shock activation of Drosophila heat shock 70 promoter driving EGFP [52] line A is strong only at the pupal stage. "+hs" indicates heat shocked animals. As controls, transheterozygotes without heatshock (-hs) and a heatshocked wt control (wt +hs) were included. [file 1471-213X-10-53-S1.JPEG]
